# Bryophyte Community Composition and Diversity as Bioindicators of Elevational Zonation in Tropical Rainforests in Hainan Island, China

**DOI:** 10.3390/plants14203209

**Published:** 2025-10-19

**Authors:** Xin Su, Tianyun Qi, Yuanling Li, Wenjuan Wang, Donghai Li, Xiaobo Yang, Jiewei Hao

**Affiliations:** 1 School of Ecology, Hainan University, Haikou 570228, China; suxin233@hainanu.edu.cn (X.S.); 23220951320126@hainanu.edu.cn (T.Q.); wangwj@hainanu.edu.cn (W.W.); dhlye@163.com (D.L.); yanfengxb@163.com (X.Y.); 2 Hainan Academy of Forestry (Hainan Academy of Mangrove), Haikou 571924, China; liyuanlinghn@163.com; 3 International Joint Center for Terrestrial Biodiversity Around South China Sea of Hainan Province, Hainan University, Haikou 570228, China

**Keywords:** elevational zonation, bioindicators, bryophyte community, national park, tropical rainforest

## Abstract

Although mountain vertical vegetation belts are key in revealing the response to climate change and the maintenance mechanism of biodiversity, traditional field surveys and remote sensing methods face significant limitations in the structurally complex tropical humid mountainous regions of Hainan Island. As bryophytes are good microclimate indicators and characteristic components of the structure of the tropical rainforest, they may be useful tools for the construction of a general scheme of the altitudinal zonation of tropical rainforests. We surveyed bryophyte communities across eight elevations and three vegetation types at LiMu Mountain, southern China. Bryophyte species alpha diversity increased significantly as elevation increased, while beta diversity showed the contrasting pattern. Bryophyte community composition differed significantly along elevation gradients and the distribution of vegetation types was clearly distinguished by three significantly different bryophyte assemblages with specific elevational range. Hierarchical partitioning revealed that microclimate outweighed topography in structuring communities, aligning with global patterns of bryophyte thermal sensitivity. Bryophytes are effective bioindicators for tropical rainforest elevational zonation, reflecting fine-scale environmental gradients. Their sensitivity to microclimate supports their utility in monitoring vegetation shifts under climate change, particularly in topographically complex regions.

## 1. Introduction

Mountain regions, especially in the tropics, are habitats and havens for a substantial proportion of the world’s species, as well as biodiversity hotspots and conservation priority regions due to their high habitat heterogeneity and climate diversity [1,2]. However, mountain regions are more vulnerable to climate change, and the elevational spectrum of these gradients serves as a critical indicator of climate change and amplifies the signals of climate variations [3]. Therefore, accurately describing the vertical zones of mountainous vegetation types is an effective approach to unraveling the complexities and heterogeneity of mountain environments [4]. National parks, often situated in complex mountainous regions, preserve pristine natural habitats due to limited human interference. They exhibit distinct vertical vegetation zones, serving as havens for biodiversity and showcasing key ecological processes [5], and become the focus area for examining elevational belts for patterns of vegetation [6].

Prior studies on mountain vertical vegetation belts in China relied on limited field surveys or spatially/temporally discontinuous data from representative communities [7], constraining comprehensive analysis of vegetation composition, elevational belt spectrum, species diversity, and their environmental correlations [6,8]. Consequently, both traditional field methods and remote sensing exhibit limited efficacy in classifying vegetation in structurally complex humid tropical mountains [8,9,10]. Distribution patterns of mountainous vegetation types provide a theoretical basis for the conservation and utilization of mountain biodiversity. Elevation has a strong impact on vegetation structure in most mountain ecosystems worldwide. The species richness of a specific plant community clearly changes along its altitudinal distribution range. Therefore, studies on the distribution and diversity of plant communities are of paramount importance for understanding the structure and ecology of forest communities [11,12].

Bryophytes constitute an essential component of tropical forest ecosystems due to their high biodiversity [13,14], and they are widely recognized as effective biological indicators of forest integrity because of their sensitivity to environmental changes [15,16,17,18]. In humid tropical regions, mountainous vegetation displays pronounced vertical zonation, which leads to variations in internal environmental conditions and vegetation structure, thereby influencing the composition and diversity of bryophyte communities [19,20,21]. Significant differences in bryophyte diversity have been observed between the tropical lowland seasonal rainforest and the subtropical montane moist forest across different layers in Yunnan, China. Moreover, the distinction between tropical lowland cloud forests and tropical lowland rainforests can be reflected through the presence of epiphyllous species within moss communities in French Guiana [19,20].

National Park of Hainan Tropical Rainforest, located in the southern–central mountainous region of Hainan Island, is an ecological hotspot of the northern edge of the Indo-Malayan rainforest [22,23]. Although the vertical gradient of vegetation on Hainan Island has been mapped and classified into tropical lowland rainforest, tropical mountain rainforest, tropical cloud forest, and mountain shrub [8], producing a fine-scale distribution map with specific boundaries in complex mountainous areas remains a challenge due to the lack of dominant species and the existence of transitional zones [8]. In this study, we hypothesize that (1) Bryophyte species richness increases with increasing elevation. (2) Bryophyte communities can precisely indicate distinct vegetation types within the vertical zonation spectrum of tropical rainforests. (3) Microclimate and topographic factors will drive bryophyte diversity and composition across elevation gradients.

## 2. Results

### 2.1. Variation in Species Diversity Along the Elevation Gradient

**Figure 1:**
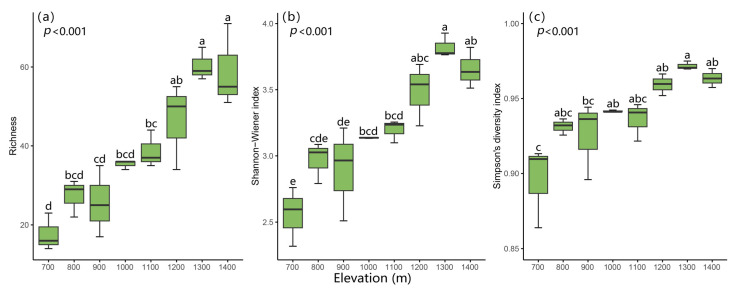
Changes in alpha diversity indices of bryophyte communities at eight elevations on LiMu Mountain, Richness (**a**), Shannon–Wiener index (**b**) and Simpson’s diversity index (**c**). Bars sharing the same letter do not differ significantly (*p* < 0.05).

**Figure A1:**
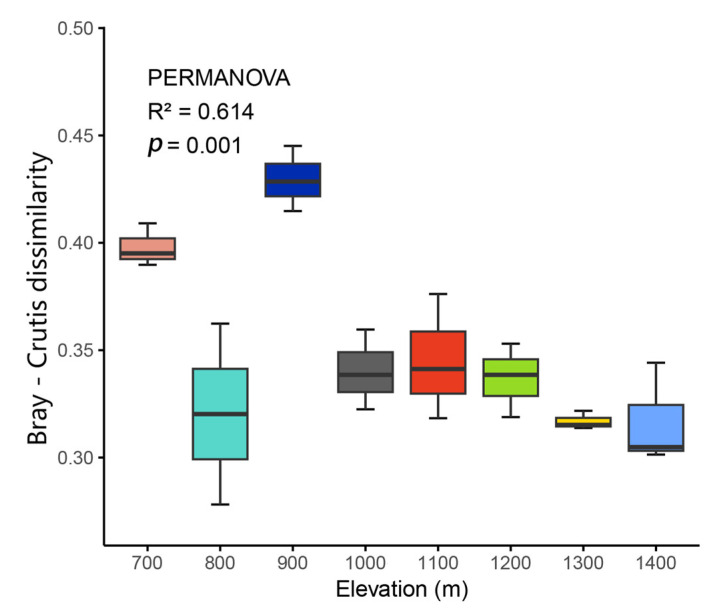
Beta diversity based on Bray-Curtis dissimilarity index assessed by PERMANOVA along altitude gradient.

A total of 195 bryophyte species were identified and assigned to 43 families and 83 genera (Table S1). Bryophyte species diversity differed significantly with elevation (Figure 1). All the three diversity indices increased along with the elevation, with minimum values obtained at 700 m. The Shannon–Wiener index and Simpson’s diversity index reached maximum values at 1300 m. The maximum richness value was obtained at 1400 m (Figure 1). Beta diversity exhibited a decreasing trend along the elevational gradient (Figure A1), although PERMANOVA showed that the difference in Bray–Curtis dissimilarity index values among bryophyte communities along the elevation gradient was significant (*p* < 0.001).

### 2.2. Community Assemblages Along the Elevation Gradient

**Figure 2:**
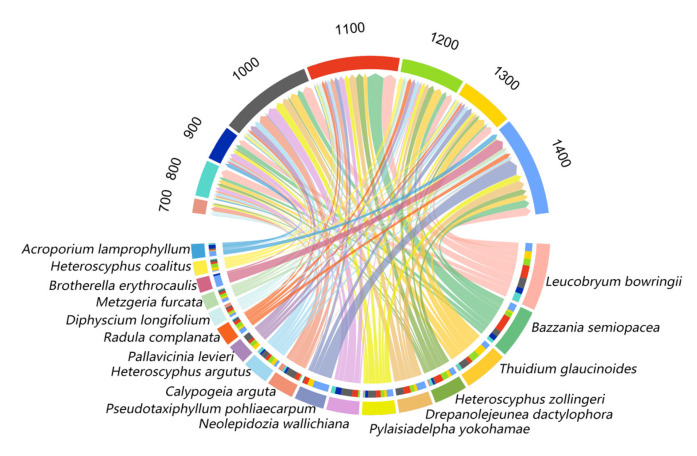
Relative frequency of dominant species of bryophytes (lower half circle) at eight altitudes (upper half circle).

The relative frequency of bryophyte species varied greatly along elevations (Figure 2). Overall, there were 17 dominant species. Among them, *Leucobryum bowringii* Mitt. (7.19%), *Bazzania semiopacea* N. Kitag. (5.34%), *Thuidium glaucinoides* Broth. (4.83%), and *Heteroscyphus zollingeri* (Gottsche) Schiffn. (3.7%) were the dominant species, accounting for 17.35% of total species. *Drepanolejeunea dactylophora* (Gottsche, Lindenb. & Nees) J.B.Jack & Steph., *Pylaisiadelpha yokohamae* (Broth.) W.R.Buck, *Neolepidozia wallichiana* (Gottsche) Fulford & J.Taylor, *Pseudotaxiphyllum pohliaecarpum* (Sull. & Lesq.) Z.Iwats., *Calypogeia arguta* Nees & Mont., *Heteroscyphus argutus* (Reinw., Blume & Nees) Schiffn., *Pallavicinia levieri* Schiffn., *Radula complanata* (L.) Dumort., *Diphyscium longifolium* Griff., *Metzgeria furcata* (L.) Corda, *Brotherella erythrocaulis* (Mitt.) M.Fleisch., *Heteroscyphus coalitus* (Hook.) Schiffn., and *Acroporium lamprophyllum* Mitt. were the common species, accounting for 30.88% of the total, and together with the dominant species, they constituted the main bryophyte species on LiMu Mountain.

**Figure 3:**
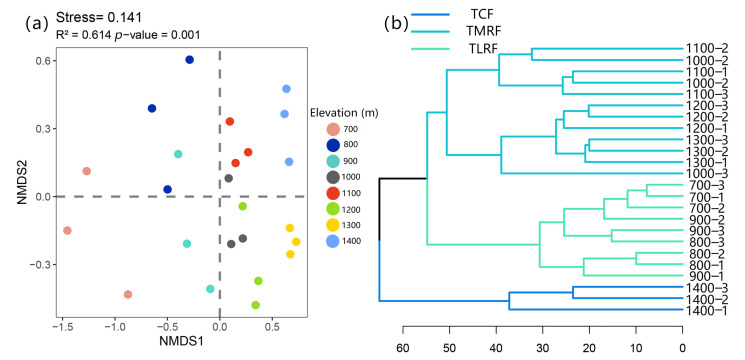
Non-metric multidimensional scaling (NMDS) ordination plot based on the Bray–Curtis dissimilarity index of bryophyte communities along elevation gradients (**a**) and cluster analysis of bryophytes based on plot-level data (**b**).

**Figure A2:**
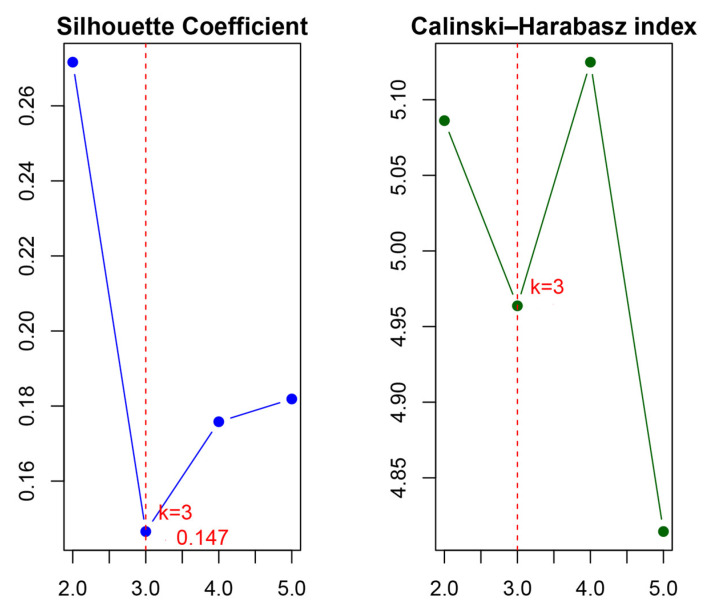
Classification of bryophyte communities using cluster analysis with Euclidean distance and Ward’s minimum variance method, where the optimal number of clusters was determined by evaluating the Silhouette width and Calinski-Harabasz index across k values.

According to the results of NMDS, Bryophyte species composition differed significantly between the eight elevations and was greatly influenced by elevation (stress = 0.141, R^2^ = 0.614, *p* = 0.001; Figure 3a). NMDS axis 1 partitioned the high-elevation region with the low- and middle-elevation regions, recognizing that bryophyte community composition in the former region differs from the latter (Figure 3a). We clearly distinguished the distribution pattern of three vegetation types by categorizing the eight bryophyte communities into three assemblages through cluster analysis (Figure 3b). Additionally, the tropical lowland rainforest (TLRF) ranges from 700 to 900 m, while tropical mountain rainforest (TMRF) spans between 1000 and 1300 m, and tropical cloud forest is located above 1400 m, with the zones from 900 to 1000 m and from 1300 to 1400 m acting as transition areas (ecotone). The bryophyte alpha diversity, including richness; Shannon–Wiener index; and Simpson’s diversity index of three different vegetation types were clearly different (Figure A2).

### 2.3. Local Indicator Species for Vegetation Types Along Elevation

The IndVal analyses showed that 27 species, including 15 mosses and 12 liverworts, acted as local indicators for these three vegetation types: tropical lowland rainforest (TLRF), tropical mountain rainforest (TMRF), and tropical cloud forest (TCF) (Indval > 60%, *p* < 0.05, Table 1). Two moss species, *Leucobryum chlorophyllosum* Müll.Hal., and *Isocladiella surcularis* (Dixon) B.C.Tan & Mohamed, were specific to TLRF, while three liverworts, *Riccardia plumosa* (Mitt.) E.O.Campb., *Pallavicinia levieri* Schiffn., and *Heteroscyphus coalitus* (Hook.) Schiffn., and one moss species, *Distichophyllum mittenii* Bosch & Sande Lac., were distinct indicators for TMRF. There were 21 species, consisting of 12 mosses and 9 liverworts, exclusive to TCF. This included 1 moss *Brotherella fauriei* (Cardot) Broth. and 1 liverwort *Plagiochila peculiaris* Schiffn., with Indval being 1.

### 2.4. Indicator Species for Vegetation Types Along Elevation

**Figure 4:**
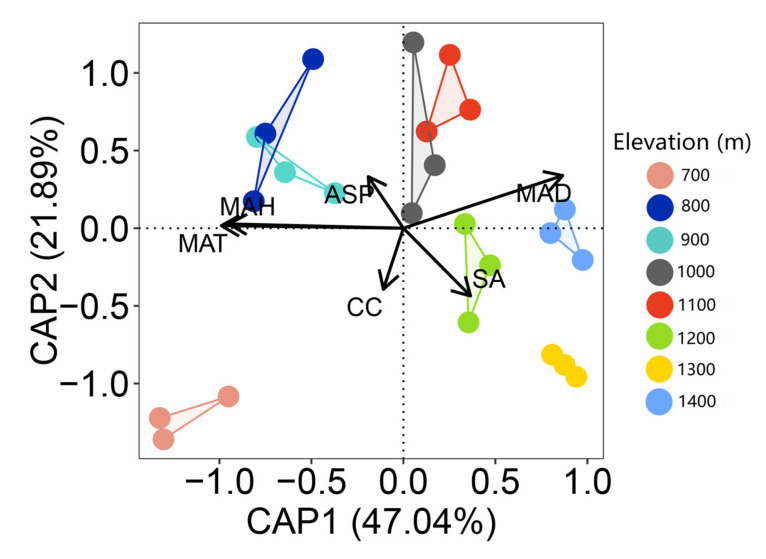
Distance-based redundancy analysis (db-RDA) identifying the relationships between bryophyte communities and environmental factors at eight elevations.

**Figure 5:**
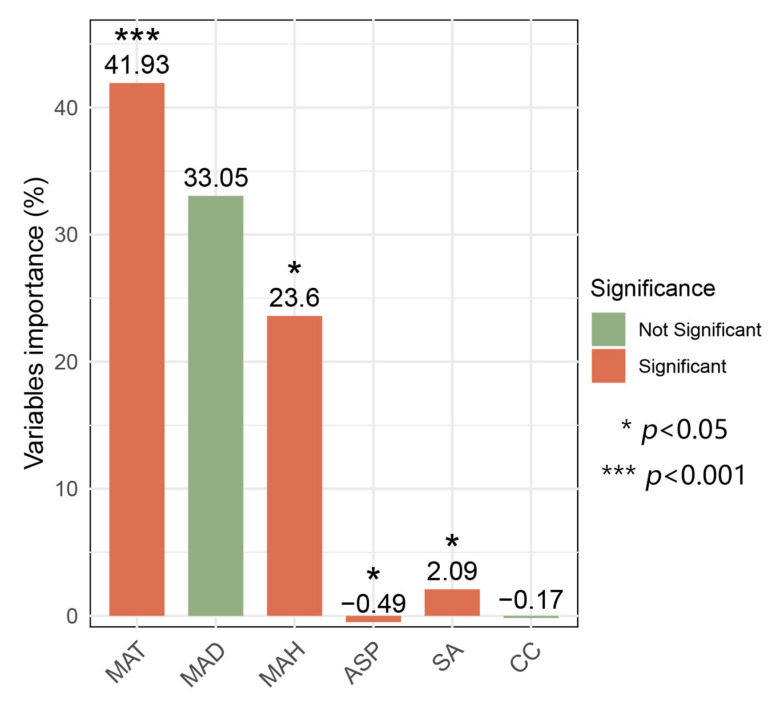
Hierarchical partitioning displaying the environment variables’ importance in bryophyte communities at eight elevations.

The first two axes of the db-RDA results explained 68.93% of the variance in bryophyte communities, and the community composition shows significant differentiation along the first ordination axis, reflecting the distribution pattern of the moss community composition along the elevation gradient (F = 2.44, *p* = 0.001; Figure 4). Among numerous environmental factors, mean annual relative humidity (MAH), mean annual temperature (MAT), and mean annual dew point temperature (MAD) have the most crucial influence on the elevation gradient pattern of the bryophyte community composition. Slope angle (SA), aspect (ASP), and mean annual relative humidity (MAH) correlated positively with bryophyte communities at higher elevation (1000 m–1400 m) and were negatively linked with lower elevations (700 m–900 m). Mean annual temperature (MAT), mean annual dew point temperature (MAD), and canopy closure (CC) positively were associated with bryophyte communities at lower elevations (700 m–900 m), and they were negatively associated with higher elevation (1000 m–1400 m). Hierarchical partitioning results showed three factors had a significant individual contribution to the explained variation among bryophyte communities, with MAT having the highest individual contribution (41.93%), followed by MAH (23.6%) and SA (2.09%) (Figure 5).

## 3. Discussion

### 3.1. Species Diversity and Distribution Patterns of Bryophyte Communities Along the Elevation

In the present study, a large number of bryophyte species (195) were identified in LiMu Mountain, representing more than a quarter of the total species pool of bryophytes in Hainan Island [24]; additionally, the interesting point is that species richness of liverworts surpasses that of mosses. The total bryophyte species number is greater than that of tropical regions, e.g., the 166 bryophyte species of Baru Volcano National Park, western Panama [25], while it is lower than 254 species of Marojejy National Park, northeastern Madagascar [26]. The species richness of liverworts surpasses that of mosses, which is in line with previous studies in Asian and African tropics characterizing tropical rainforest climate [27]. As the study by Wang et al. and Iskandar et al. [28,29] stated, the highest richness of liverworts appears in the wet tropic regions, especially in the high mountains featuring diverse habitat heterogeneity and climate diversity (like precipitation-related factors). The results indicate that although Hainan Island is located at the northern edge of the Indo-Malayan rainforest [22], it is an integral part of a global biodiversity hotspot area, whose biodiversity conservation priority should be considerably emphasized [30].

Our results showed that bryophyte alpha diversity exhibited an increasing pattern with increasing elevation, while beta diversity showed a contrasting pattern, which contributes to the understanding of the spatial organization of bryophyte diversity at different scales [31]. For alpha diversity, the same pattern was also found in tropical African, La Réunion, Mascarene archipelago [32]. A similar pattern of elevational diversity was also observed in subtropical regions [33]. However, a hump-shaped pattern, with a peaking near-mountain climax, was observed for moss species along the elevational gradient on the Jianfengling, ranging from 110 m to 1390 m in southwestern Hainan Island, China [34]. A hump-shaped pattern with a mid-elevation peak (increasing beta diversity pattern) was described for bryophyte species on a subtropical oceanic Island (La Palma, Canary Islands) [31]. Other distribution patterns, such as a decreasing pattern in Uganda [35], or no obvious trend [36,37] were also detected. The scale effect might explain the difference between these alpha patterns. When the elevation gradient is relatively long, a hump-shaped distribution will occur instead of a linear pattern [26,34,38]. If the elevational gradient is relatively short or only part of the whole mountain gradient, then a linear pattern rather than a hump-shaped pattern would be observed, like the study conducted on La Réunion, Mascarene archipelago, with an elevation gradient from 250 m to 850 m [32], and the present study, with a gradient ranging from 700 to 1400 m. In addition, a decline in species richness would appear towards lower elevations due to the increasingly dry and hot climate. Such conditions are generally unfavorable for bryophytes and result in low bryophyte richness, as observed during casual observations [39,40]. Most bryophytes prefer to live in humid conditions [41]; this explains why bryophyte diversity increased with increases in humidity and peaked at elevations with optimal humid conditions [26,42]. The higher beta diversity at lower elevations may result from the complex habitat and more diverse disturbances; however, the habitats are relatively stable and homogeneous, making the community composition more consistent at middle and high elevations [43]. The densely distributed bamboos at 700 m and historical extensive shifting cultivation and logging in the lowlands [44] might result in relatively great heterogeneity and favor the presence of more and different species, even with lower species alpha diversity.

### 3.2. Bryophyte Communities Coupling with Vegetation Types Along Elevation Gradients

NMDS analysis showed that bryophyte species composition differed significantly between the eight elevations. Specifically, bryophyte community composition in the high-elevation regions differed significantly from middle and low elevations as shown in Figure 3a. In the present study, the sampling sites in the high-elevation region are characterized by heavy precipitation, frequent fogs, and high winds, which led to significant differences in bryophyte community structure at high elevations from other elevation gradients. This indicates that water availability (e.g., rain, fog) is essential for the distribution of bryophyte species, as shown by many authors [26,45]. In addition to the direct effects of climate on bryophyte performance, the changing structure and composition of the forest affects the microclimate at ground level (not only temperature and humidity, but also, e.g., light levels and wind speeds), therefore changing the bryophyte community structure [20,46]. Also, indicator species analyses showed that each vegetation type was characterized by unique taxa as shown in Table 1. As tropical cloud forests (TCFs) are one of the world’s most species- and endemism-rich terrestrial ecosystems, 21 species were specific to tropical cloud forest in the present study. These can serve as early-warning markers for cloud forest boundary shifts under climate change [16]. Here, we clearly distinguished the distribution pattern of three vegetation types by categorizing the eight bryophyte communities into 3 assemblages as present in Figure 3b. On LiMu Mountain, the tropical lowland rainforest (TLRF) ranges from 700 to 900 m, while tropical mountain rainforest (TMRF) spans between 1000 and 1300 m, and tropical cloud forest is located above 1400 m. It is evident that the diversity distribution pattern of bryophytes not only monotonically correlated with the elevation, but was also in a coupling relationship with vertical vegetation zones, demonstrating that bryophytes are useful tools for scheming the elevational zonation of tropical rainforest along complex mountainous region in humid tropics. The present study affirmed the previous discoveries that bryophytes are good habitat indicators and exhibit a distinct connection with diverse forest types [47]. Notably, non-vascular plants are often overlooked in vegetation assessments despite their indicator value. Given that LiMu Mountain is relatively low (maximum elevation 1411.7 m) and has a well-documented history of shifting cultivation and logging in the lowlands, the intrinsic linkage between bryophytes and vegetation types should be further tested in high-mountain primary tropical rainforests such as Yinggeling or Wuzhi Mountain [48].

### 3.3. The Contribution of Environmental Factors to Bryophyte Distribution

Bryophyte communities were significantly influenced by understory microclimate (MAT, MAD, and MAH) and topographic factors like ASP and SA jointly. Additionally, hierarchical partitioning analysis showed that MAT, MAH, and SA were the first three primary factors influencing species distribution pattern, and temperature-related factors, such as MAT and MAH, had the largest impact on the forest bryophyte diversity and distribution in the lowland. Climatic characteristics are among the key drivers of bryophyte community variation with elevation [25,49], which is also demonstrated by other researchers [26,37]. As bryophytes are sensitive to environmental climate, richness increases with increasing habitat quality, and then declines as habitats become more stressed [36]. As we mentioned above, at lower elevations with high temperatures, when the humidity continuously increases, the bryophyte diversity will decrease within the studied lowland rainforest sites, where the lower elevation (700 m~900 m) combined with higher humidity and higher temperatures tend to be associated with significant vascular plants, including woody plants and herbaceous plants. These herbaceous plants will compete for light, space, water, and nutrients with ground bryophytes [50], whereas ground bryophytes are less competitive due to their smaller size and absence of vascular tissue. In addition, the abundant woody plants will lead to limited space and light penetration rates and precipitation for understory plants [51]. MAH had the greatest impact on the forest bryophyte diversity and distribution at high elevation. As elevation increases, habitats at higher elevations are turned into cooler climatic conditions (e.g., low temperatures, high humidity, and sufficient precipitation) that may lead to higher bryophyte diversity [26,42]. Our results show an increasing trend in the diversity of exclusive bryophytes in different vegetation types with increasing elevation, suggesting that the increment of some species (such as liverworts) due to favorable climatic conditions may be a cause of the increase pattern [52].

Although bryophytes are adapted to relatively high temperatures, the increasing frequency and intensity of heatwaves, which have become more common and are projected to intensify under ongoing climate change, will affect the richness and colonization of bryophytes in tropical mid- to low-altitude regions [17,34]. To adapt to global warming, the range of species tends to shift toward the poles or higher altitudes, while increasing temperature might lead to radical changes at a dominant vegetation in larger scale. Bryophyte communities couple with vegetation types along Limu Mountain, suitable microhabitats inhabited by many bryophyte species might still persist and enable them to survive within their original distribution range or at least in parts of it. In the long run, the conditions of these microhabitats will gradually deteriorate [17,53], and bryophytes might be negatively affected or even be at risk of extinction.

## 4. Materials and Methods

### 4.1. Study Site

**Figure 6:**
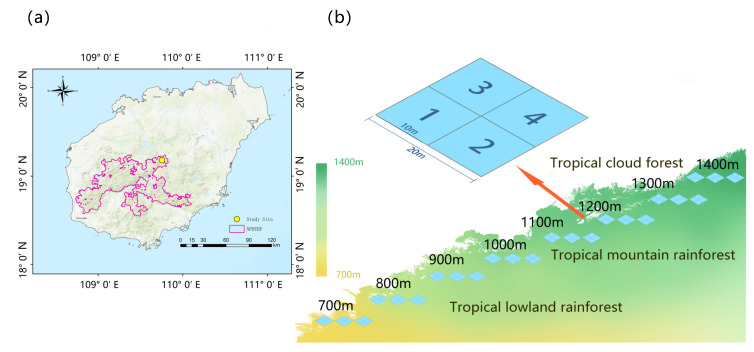
Schematic map of sampling locations (**a**); sampling plots (**b**) along elevation gradient and vegetation types in LiMu Mountain.

LiMu Mountain, located in LiMu Mountain Branch of the National Park of Hainan Tropical Rainforest—an area where primary natural habitats are well preserved—was selected as the study area (19°07′22″—19°14′03″ N, 109°39′05″—109°48′31″ E) (Figure 6a). LiMu Mountain (main peak) is one of the well-protected regions with tropical rainforest as the typical vegetation. It represents an elevational gradient from 650 m to 1412 m above sea level. This area has a tropical climate dominated by the summer monsoon with a dry season extending between November and April and a rainy season between May and October [54]. The average annual rainfall is over 1600 mm, but this is not evenly distributed because of the orography of the island. The eastern section of the island receives an annual average precipitation of 2000–2400 mm, whilst in the western sector, this value is reduced to 1000–2000 mm [27]. During the rainy season, the island is in the path of typhoons. The parent material is granite and the soil is latosol at lower elevations and mountain lateric soil at higher elevations [44].

### 4.2. Bryophyte Sampling Collection and Identification

Three 20 × 20 m plots were established at each elevation (100 m apart for 700 m, 800 m, 900 m, 1000 m, 1100 m, 1200 m, 1300 m, and 1400 m) on the southern slope of LiMu Mountain in April 2023. To account for microhabitat heterogeneity, all three plots were homogenous and comparable to each other regarding vegetation type (according to typical representative vascular species), canopy height and microclimate, and they were set up 10–15 m away from the trail at a gentle slope for convenient access. All plots were set 50 m apart to ensure the independence of each plot within the same elevation in the natural forest ecosystems. At each elevation, 20 × 20 m was divided into four 10 × 10 m subplots, from which all bryophyte samples growing on soil, rock, living leaf, tree base and dead wood were collected, with a total of 240 samples (5 substrates × 4 replicates× 4 subplots × 3 plots). The specimens were separated from different plants by examining morphological characteristics using a SZ745 stereo microscope from LIOO (Germany). Subsequently, all specimens for anatomical characters were identified to species level and photographed under a light microscope (ZEISS Primostar 3, Carl Zeiss AG, Oberkochen, Baden-Württemberg, Germany) equipped with a digital camera (TouPTek, IUA2300KPA, Hangzhou, China). Classification and identification were mainly based on Flora Bryophytorum Sinicorum and Chinese illustrated Bryophytes. The full species list from all elevations is given in Table S1.

### 4.3. Environmental Variables

To identify the potential drivers of species diversity and composition of bryophyte communities along the elevational gradients, environmental data like air temperature, relative humidity, dew point temperature, and other parameters were obtained. Three data-loggers (HOBO MX2301A, Onset, Bourne, MA, USA, one in each plot) were set up from April 2023 to collect air temperature, dew point, and relative humidity at each elevation. Mean annual temperature (MAT), mean annual relative humidity (MAH), and mean annual dew point (MAD) were computed by obtained data using a data-logger (HOBO MX2301A). Z-score was applied to standardize the environmental data, including slope angle (SA), aspect (ASP), and canopy closure (CC). To avoid the deviation of the circular dimension of aspect from the sorting results, the original aspect (0–360°) was decomposed into two linear variables, the northward degree (cosine-transformed aspect) and the eastward degree (sine-transformed aspect), through trigonometric functions before being included in the RDA analysis [55]. Geographic location was recorded at each sampling site [56]. Solar radiation (SR), wind speed (WS), and annul precipitation (AP) at each elevation were downloaded from the WorldClim database with a resolution of approximately 1 km. Data were extracted for each elevation in ArcGIS 10.2 (Edlands, ESRI, CA, USA).

### 4.4. Statistical Analyses

All statistical analyses were conducted in R (v.4.4.1; http://www.r-project.org/ (accessed on 4 November 2024)). We calculated richness, Shannon–Wiener index, Simpson’s diversity index, and β diversity based on the relative abundance of species data (Bray–Curtis dissimilarity index) to explore the elevational diversity distribution patterns of bryophyte community. All models met the assumptions of the normality of residuals and homogeneity of variances, and the above analyses were performed using the “vegan” packages (2.6-8) [57]. Additionally, the significance of differences in β diversity (based on standardized relative abundance data using Bray–Curtis dissimilarity) among the eight elevations was assessed using PERMANOVA to evaluate variation across elevation gradients.

To visualize variations in bryophyte assemblages and to examine the elevational differences in compositional dissimilarities, non-metric multidimensional scaling (NMDS) ordinations based on Bray–Curtis dissimilarity and PERMANOVA were performed in the R package “vegan” (2.6-8) [58]. Cluster analysis at the plot level was performed to classify bryophyte communities into distinct vegetation types in an objective and independent manner. The clustering process utilized Euclidean distance in combination with Ward’s minimum variance method. The optimal number of clusters was determined through a comprehensive evaluation of the Silhouette width and the Calinski–Harabasz index across k value [57]. Indicator species analyses were conducted to identify representative species among all vegetation types [59]. Higher indicator values suggest that a species occurs more frequently and is more specific to a location, and this species can be regarded as highly specific to the particular area. Indicator analyses were performed with the “indval” function implemented in the R package “labdsv” [60]. Distance-based redundancy analysis (db-RDA) using Monte Carlo permutation (999 repetitions) was conducted to determine the main environmental factors influencing bryophyte communities based on Hellinger pre-transformed community data, which was performed in the “rdacca.hp” package [61,62,63]. 

## 5. Conclusions

We conclude that plant diversity should be studied at multiple scales. Cluster analysis indicates that bryophyte communities are independently classified into vegetation types defined by vascular plants and show the specific elevational range for each elevational zone of tropical rainforest. Our study shows that bryophytes are good indicators of habitat conditions and intrinsically show associations with different forest types. Bryophytes are useful tools for scheming the elevational zonation of tropical rainforests on Hainan Island. Microclimate exerted a stronger influence than all other environmental variables on bryophyte community across elevation gradients. Such information not only provides further insight into the effects of elevation and vegetation types on biodiversity patterns, but is also essential for predicting the impact of future climate change. Additionally, further study is necessitated due to the relatively small altitude range and impact of historical forest transformations in lower areas.

## Figures and Tables

**Table 1:** Percentage indicator values (IndVal > 60%) of bryophyte species for vegetation types.

Vegetation Type	Indicator Species	% IndVal	*p* Value
TLRF	*Isocladiella surcularis* (Dixon) B.C.Tan & Mohamed	68.13%	0.005
	*Leucobryum chlorophyllosum* Müll.Hal.	66.40%	0.012
TMRF	*Riccardia plumosa* (Mitt.) E.O.Campb.	79.67%	0.002
	*Pallavicinia levieri* Schiffn.	73.34%	0.019
	*Heteroscyphus coalitus* (Hook.) Schiffn.	63.99%	0.009
	*Distichophyllum mittenii* Bosch & Sande Lac.	62.50%	0.032
TCF	*Plagiochila peculiaris* Schiffn.	100.00%	0.002
	*Brotherella fauriei* (Cardot) Broth.	100.00%	0.002
	*Brotherella erythrocaulis* (Mitt.) M.Fleisch.	95.00%	0.001
	*Leucobryum scabrum* Sande Lac.	87.42%	0.001
	*Plagiochila trabeculata* Steph.	86.02%	0.001
	*Pseudotaxiphyllum pohliaecarpum* (Sull. & Lesq.) Z.Iwats.	85.04%	0.001
	*Leucoloma molle* (Müll. Hal.) Mitt.	82.76%	0.003
	*Lejeunea alata* Gottsche	81.99%	0.001
	*Sematophyllum subpinnatum* (Brid.) E.Britton	80.00%	0.002
	*Leucobryum boninense* (Müll.Hal.) A.Jaeger	78.05%	0.004
	*Pyrrhobryum latifolium* Mitt.	71.49%	0.007
	*Thuidium pristocalyx* (Müll.Hal.) A.Jaeger	68.97%	0.005
	*Acroporium lamprophyllum* Mitt.	68.35%	0.017
	*Hookeriopsis utacamundiana* (Mont.) Broth.	66.67%	0.01
	*Radula cavifolia* Hampe	66.67%	0.01
	*Frullania linii* S.Hatt.	66.67%	0.013
	*Cololejeunea ceratilobula* (P.C.Chen) R.M Schust.	63.16%	0.004
	*Dicranoloma dicarpum* (Nees) Paris	62.75%	0.008
	*Drepanolejeunea dactylophora* (Gottsche, Lindenb. & Nees) J.B.Jack & Steph.	62.52%	0.025
	*Metalejeunea cucullata* (Reinw., Blume & Nees) Grolle	62.07%	0.022
	*Cololejeunea longifolia* (Mitt.) Benedix	61.54%	0.013

**Figure A3:**
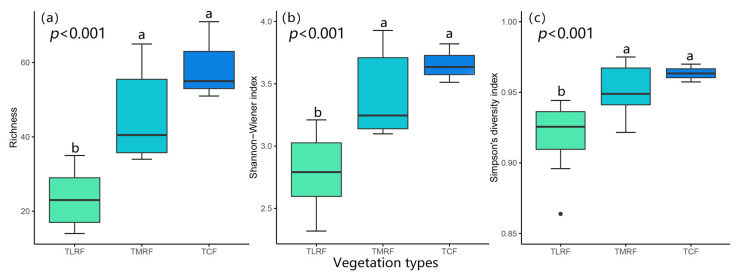
Changes in Richness (**a**), Shannon–Wiener index (**b**), and Simpson’s diversity index (**c**) of bryophytes among tropical lowland rainforest (TLRF), tropical mountain rainforest (TMRF), and tropical cloud forest (TCF) along the LiMu Mountain. Error bars depict standard error of the means. Bars with different letters are significantly different.

## Data Availability

Data available in article Supplementary Materials.

## Supplementary Materials

The following supporting information can be downloaded at: https://www.mdpi.com/article/10.3390/plants14203209/s1, Table S1: A total of 195 bryophyte species were identified in Mt LiMu; Table S2: Dominant species of bryophytes at each altitudinal gradient.
