# Supplementary material for: Bryophyte Community Composition and Diversity as Bioindicators of Elevational Zonation in Tropical Rainforests in Hainan Island, China
Source: Plants (Basel). 2025 Oct 19;14(20):3209. doi: 10.3390/plants14203209 (PMC12566818; doi:10.3390/plants14203209)
Supplement: Supplementary file 1 [file plants-14-03209-s001.zip › plants-3827342-supplementary.pdf]

Supporting information to the paper

Table S1. A total of 195 bryophytes species were identified in Mt LiMu.

Family	Genus	Species
Aneuraceae	*Aneura*	*Aneura pinguis* (L.) Dumort.
	Riccardia	*Riccardia plumosa* (Mitt.) E. O. Campb.
		Riccardia submultifida Horik.
Blepharostomataceae	*Blepharostoma*	*Blepharostoma trichophyllum* (L.) Dumort.
Brachytheciaceae	*Brachythecium*	*Brachythecium buchananii* (Hook.) A. Jaeger
	Rhynchostegiella	*Rhynchostegiella leptoneura* Dixon & Thér.
	Rhynchostegium	*Rhynchostegium pallenticaule* Müll. Hal.
		Rhynchostegium subspeciosum (Müll. Hal.) Müll. Hal.
	Palamocladium	*Palamocladium leskeoides* Britton
Bryaceae	*Bryum*	*Bryum pallescens* Schleich. ex Schwägr.
		Bryum coronatum Schwägr.
Calymperaceae	*Calymperes*	*Calymperes serratum* A. Braun ex Müll. Hal.
		Calymperes levyanum var. *hainanense* W. D. Reese & P. J. Lin
		Calymperes fasciculatum Dozy & Molk.
	Exostratum	*Exostratum blumei* (Nees ex Hampe) L. T. Ellis
	Syrrhopodon	*Syrrhopodon prolifer* Schwägr.
		Syrrhopodon semiliber (Mitt.) Besch.
		Syrrhopodon parasiticus (Sw. ex Brid.) Besch.
		Syrrhopodon japonicus (Besch.) Broth.
		Syrrhopodon gardneri (Hook.) Schwägr.
Calypogeiaceae	*Calypogeia*	*Calypogeia arguta* Nees & Mont.
		Calypogeia tosana (Steph.) Steph.
Cephaloziaceae	*Cephalozia*	*Cephalozia gollanii* Steph.
	Nowellia	*Nowellia curvifolia* (Dicks.) Mitt.
	Schiffneria	*Schiffneria hyalina* Steph.
Cephaloziellaceae	*Cephaloziella*	*Cephaloziella microphylla* (Steph.) Douin
Daltoniaceae	*Calyptrochaeta*	*Calyptrochaeta ramosa* subsp. *spinosa* (Nog.) B. C. Tan & P. J. Lin
	Distichophyllum	*Dicranoloma dicarpum* (Nees) Paris
Dicranaceae	*Dicranoloma*	*Dicranum psathyrum* Klazenga
	Dicranum	*Holomitrium densifolium* (Wilson) Wijk & Marg.
	Holomitrium	*Leucoloma molle* (Müll. Hal.) Mitt.
	Leucoloma	*Diphyscium longifolium* Griff.
Diphysciaceae	*Diphyscium*	*Entodon longifolius* (Müll. Hal.) A. Jaeger
Entodontaceae	*Entodon*	*Fissidens pellucidus* Hornsch.
Fissidentaceae	*Fissidens*	*Fissidens guangdongensis* Z. Iwats. & Z. H. Li

		Fissidens incognitus Gangulee
		Fissidens taxifolius Hedw.
		Fissidens ganguleei Nork. ex Gang.
		Fissidens serratus Müll. Hal.
		Fissidens polypodioides Hedw.
		Fissidens schwabei Nog.
		Fissidens wichurae Broth. & M. Fleisch.
		Fissidens javanicus Dozy & Molk.
		Frullania punctata Reimers
Frullaniaceae	*Frullania*	*Frullania motoyana* Steph.
		Frullania apiculata (Reinw. Blume & Nees) Nees
		Frullania moniliata (Reinw. Blume & Nees) Nees
		Frullania linii S. Hatt.
		Haplomitrium mnioides (Lindb.) R. M. Schust.
Haplomitriaceae	*Haplomitrium*	*Herbertus dicranus* (Taylor) Trevis.
Herbertaceae	*Herbertus*	*Hookeria acutifolia* Hook. & Grev.
Hookeriaceae	*Hookeria*	*Pseudotaxiphyllum pohliaecarpum* (Sull. & Lesq.) Z. Iwats.
Hypnaceae	*Pseudotaxiphyllum*	*Ectropothecium obtusulum* (Cardot) Z. Iwats.
	Ectropothecium	*Lopidium struthiopteris* (Brid.) M. Fleisch.
Hypopterygiaceae	*Lopidium*	*Jubula japonica* Steph.
Jubulaceae	*Jubula*	*Cololejeunea inflata* Steph.
Lejeuneaceae	*Cololejeunea*	*Acrolejeunea sandvicensis* (Gottsche) Steph.
	Acrolejeunea	*Cheilolejeunea xanthocarpa* (Lehm. & Lindenb.) Malombe
	Cheilolejeunea	*Cheilolejeunea trapezia* (Nees) Mizut.
		Cheilolejeunea falsinervis Kachroo & R. M. Schust.
		Cololejeunea spinosa (Horik.) Pandé & R. N. Misra
	Cololejeunea	*Cololejeunea planissima* (Mitt.) Abeyw.
		Cololejeunea gottschei (Steph.) Mizut.
		Cololejeunea hainanensis R. L. Zhu
		Cololejeunea pseudocristallina P. C. Chen & P. C. Wu
		Cololejeunea ocellata (Horik.) Benedix
		Cololejeunea longifolia (Mitt.) Benedix
		Cololejeunea haskarliana (Lehm. & Lindenb.) Schiffn.

		Cololejeunea floccosa (Lehm. & Lindenb.)

		Schiffn.
		Cololejeunea tenella Benedix
		Cololejeunea pseudoschmidtii Tixier
		Cololejeunea raduliloba Steph.
		Cololejeunea equialbi Benedix
		Cololejeunea schwabei Herzog
		Cololejeunea ceratilobula (P. C. Chen) R. M. Schust.
		Cololejeunea wightii Steph.
		Cololejeunea rotundilobula (P. C. Wu & P. J. Lin) Piippo
		Drepanolejeunea dactylophora (Gottsche Lindenb. & Nees) J. B. Jack & Steph.
	Drepanolejeunea	*Drepanolejeunea levicornua* Steph.
		Drepanolejeunea pentadactyla (Mont.) Steph.
		Drepanolejeunea foliicola Horik.
		Drepanolejeunea spicata (Steph.) Grolle & R. L. Zhu
		Lejeunea flava (Sw.) Nees
	Lejeunea	*Lejeunea neelgherriana* Gottsche
		Lejeunea alata Gottsche
		Lejeunea eifrigii Mizut.
		Lejeunea parva (S. Hatt.) Mizut.
		Lejeunea tuberculosa Steph.
		Leptolejeunea elliptica (Lehm. & Lindenb.) Steph.
	Leptolejeunea	*Leptolejeunea apiculata* (Horik.) S. Hatt.
		Lopholejeunea subfusca (Nees) Schiffn.
		Lopholejeunea nigricans (Lindenb.) Steph.
		Lopholejeunea ceylanica Steph.
		Metalejeunea cucullata (Reinw. Blume & Nees) Grolle
	Metalejeunea	*Microlejeunea ulicina* (Taylor) Steph.
	Microlejeunea	*Spruceanthus polymorphus* (Sande Lac.) Verd.
	Spruceanthus	*Spruceanthus semirepandus* (Nees) Verd.
		Bazzania debilis N. Kitag.
Lepidoziaceae	*Bazzania*	*Bazzania fauriana* (Steph.) S. Hatt.
		Bazzania himlayana (Mitt.) Schiffn.
		Bazzania mayabarae S. Hatt.
		Bazzania semiopacea N. Kitag.
		Bazzania tridens (Reinw. Blume & Nees) Trevis.
		Kurzia gonyotricha (Sande Lac.) Grolle
	Kurzia	*Kurzia makinoana* (Steph.) Grolle

		Kurzia sinensis K. C. Chang
		Lepidozia reptans (L.) Dumort.
	Lepidozia	*Lepidozia sandvicensis* Lindenb.
		Lepidozia trichodes (Reinw. Blume & Nees) Gottsche
		Lepidozia vitrea Steph.
		Leucobryum aduncum Dozy & Molk.
	Leucobryum	*Leucobryum aduncum* var. *scalare* (Müll. Hal. ex M. Fleisch.) A. Eddy
		Leucobryum boninense Sull. & Lesq.
		Leucobryum bowringii Mitt.
		Leucobryum chlorophyllosum Müll. Hal.
		Leucobryum glaucum (Hedw.) Ångstr.
		Leucobryum javense (Brid.) Mitt.
		Leucobryum scabrum Sande Lac.
		Neolepidozia wallichiana (Gottsche) Fulford & J. Taylor
	Neolepidozia	*Zoopsis liukiuensis* Horik.
	Zoopsis	*Leucomium strumosum* (Hornsch.) Mitt.
Leucomiaceae	*Leucomium*	*Chiloscyphus aposinensis* Piippo
Lophocoleaceae	*Chiloscyphus*	*Heteroscyphus lophocoleoides* S. Hatt.
	Heteroscyphus	*Heteroscyphus zollingeri* (Gottsche) Schiffn.
		Heteroscyphus planus (Mitt.) Schiffn.
		Heteroscyphus coalitus (Hook.) Schiffn.
		Heteroscyphus argutus (Reinw. Blume & Nees) Schiffn.
		Leptobryum pyriforme Wilson
Meesiaceae	*Leptobryum*	*Chrysocladium retrorsum* (Mitt.) M. Fleisch.
Meteoriaceae	*Chrysocladium*	*Metzgeria furcata* (L.) Corda
Metzgeriaceae	*Metzgeria*	*Plagiomnium succulentum* T. J. Kop.
Mniaceae	*Plagiomnium*	*Orthomnion dilatatum* P. C. Chen
Neckeraceae	*Homaliodendron*	*Homaliodendron scalpellifolium* (Mitt.) M. Fleisch.
Orthotrichaceae	*Schlotheimia*	*Schlotheimia grevilleana* Mitt.
Pallaviciniaceae	*Pallavicinia*	Pallavicinia *lyellii* (Hook.) Gray
		Pallavicinia levieri Schiffn.
Pilotrichaceae	*Hookeriopsis*	*Hookeriopsis utacamundiana* (Mont.) Broth.
	Callicostella	*Callicostella papillata* (Mont.) Mitt.
	Actinodontium	*Actinodontium rhaphidostegum* (Müll. Hal.) Bosch & Sande Lac.
Plagiochilaceae	*Chiastocaulon*	*Chiastocaulon oppositum* (Reinw. Blume & Nees) S. D. F. Patzak M. A. M. Renner Schäf.-Verw. & Heinrichs
	Plagiochila	*Plagiochila sciophila* Nees ex Lindenb.

		Plagiochila peculiaris Schiffn.
		Plagiochila bantamensis (Reinw. Blume & Nees) Mont.
		Plagiochila fordiana Steph.
		Plagiochila junghuhniana Sande Lac.
		Plagiochila corticola Steph.
		Plagiochila trabeculata Steph.
		Plagiochila fruticosa Mitt.
Polytrichaceae	*Pogonatum*	*Pogonatum inflexum* (Lindb.) Sande Lac.
	Polytrichum	*Polytrichum commune* Hedw.
Pylaisiadelphaceae	*Brotherella*	*Brotherella erythrocaulis* (Mitt.) M. Fleisch.
		Brotherella fauriei (Cardot) Broth.
		Brotherella henonii (Duby) M. Fleisch.
		Brotherella falcata (Dozy & Molk.) M. Fleisch.
	Gammiella	*Gammiella tonkinensis* (Broth. & Paris) B. C. Tan
	Pylaisiadelpha	*Pylaisiadelpha yokohamae* (Broth.) W. R. Buck
	Taxithelium	*Taxithelium lindbergii* (A. Jaeger) Renauld & Cardot
		Taxithelium oblongifolium (Sull. & Lesq.) Z. Iwats.
Radulaceae	*Radula*	*Radula complanata* (L.) Dumort.
		Radula cavifolia Hampe
		Radula oyamensis Steph.
		Radula caduca K. Yamada
		Radula obtusiloba Steph.
		Radula philippinensis K. Yamada
		Radula apiculata Sande Lac. ex Steph.
		Radula acuminata Steph.
		Radula kojana Steph.
		Radula onraedtii K. Yamada
		Radula kurzii Steph.
		Radula madagascariensis Gottsche
		Radula japonica Gottsche ex Steph.
		Radula obscura Mitt.
		Radula formosa (C. F. W. Meissn. ex Spreng.) Nees
		Radula lindenbergiana Gottsche ex Hartm.
		Radula javanica Gottsche
Rhizogoniaceae	*Pyrrhobryum*	*Pyrrhobryum spiniforme* Mitt.
Scapaniaceae	*Scapania*	*Scapania ciliata* Sande Lac.
		Scapania ligulata subsp. *stephanii* (Müll.

		Frib.) Potemkin Piippo & T. J. Kop.
Schistochilaceae	*Schistochila*	*Schistochila aligera* (Nees & Blume) Jack & Steph.
	Acroporium	*Acroporium stramineum* (Reinw. & Hornsch.) M. Fleisch.
		Acroporium rufum (Reinw. & Hornsch.) M. Fleisch.
		Acroporium lamprophyllum Mitt.
		Acroporium secundum (Reinw. & Hornsch.) M. Fleisch.
		Acroporium diminutum (Brid.) M. Fleisch.
	Heterophyllium	*Heterophyllium affine* M. Fleisch.
	Isocladiella	*Isocladiella surcularis* (Dixon) B. C. Tan & Mohamed
	Sematophyllum	*Sematophyllum subhumile* (Müll. Hal.) M. Fleisch.
		Sematophyllum phoeniceum (Müll. Hal.) M. Fleisch.
		Sematophyllum subpinnatum (Brid.) E. Britton
	Trichosteleum	*Trichosteleum stigmosum* Mitt.
	Wijkia	*Wijkia deflexifolia* Crum
		Wijkia surcularis (Mitt.) Crum
Thuidiaceae	*Thuidium*	*Thuidium pristocalyx* (Müll. Hal.) A. Jaeger
	Thuidium	*Thuidium glaucinoides* Broth.
Trichocoleaceae	*Trichocolea*	*Trichocolea tomentella* (Ehrh.) Dumort.

Table S2. Dominant species of bryophytes at each altitudinal gradient.

Elevation (m)	Species
700	*Leucobryum bowringii* Mitt.
	Thuidium glaucinoides Broth.
	Heteroscyphus zollingeri (Gottsche) Schiffn.
	Pylaisiadelpha yokohamae (Broth.) W. R. Buck
	Neolepidozia wallichiana (Gottsche) Fulford & J. Taylor
	Calypogeia arguta Nees & Mont.
	Heteroscyphus argutus (Reinw. Blume & Nees) Schiffn.
	Diphyscium longifolium Griff.
	Metzgeria furcata (L.) Corda
	Acroporium lamprophyllum Mitt.
800	*Leucobryum bowringii* Mitt.
	Bazzania semiopacea N. Kitag.
	Thuidium glaucinoides Broth.
	Heteroscyphus zollingeri (Gottsche) Schiffn.
	Pylaisiadelpha yokohamae (Broth.) W. R. Buck
	Neolepidozia wallichiana (Gottsche) Fulford & J. Taylor
	Calypogeia arguta Nees & Mont.
	Heteroscyphus argutus (Reinw. Blume & Nees) Schiffn.
	Pallavicinia levieri Schiffn.
	Radula complanata (L.) Dumort.
	Metzgeria furcata (L.) Corda
	Heteroscyphus coalitus (Hook.) Schiffn.
	Acroporium lamprophyllum Mitt.
900	*Leucobryum bowringii* Mitt.
	Bazzania semiopacea N. Kitag.
	Heteroscyphus zollingeri (Gottsche) Schiffn.
	Drepanolejeunea dactylophora (GottscheLindenb. & Nees) J. B. Jack & Steph.
	Pylaisiadelpha yokohamae (Broth.) W. R. Buck
	Neolepidozia wallichiana (Gottsche) Fulford & J. Taylor
	Calypogeia arguta Nees & Mont.
	Heteroscyphus argutus (Reinw. Blume & Nees) Schiffn.
	Radula complanata (L.) Dumort.
	Metzgeria furcata (L.) Corda
	Brotherella erythrocaulis (Mitt.) M. Fleisch.
	Acroporium lamprophyllum Mitt.
1000	*Leucobryum bowringii* Mitt.
	Bazzania semiopacea N. Kitag.
	Thuidium glaucinoides Broth.
	Heteroscyphus zollingeri (Gottsche) Schiffn.
	Drepanolejeunea dactylophora (Gottsche Lindenb. & Nees) J. B. Jack & Steph.

	Pylaisiadelpha yokohamae (Broth.) W. R. Buck
	Neolepidozia wallichiana (Gottsche) Fulford & J. Taylor
	Pseudotaxiphyllum pohliaecarpum (Sull. & Lesq.) Z. Iwats.
	Calypogeia arguta Nees & Mont.
	Heteroscyphus argutus (Reinw. Blume & Nees) Schiffn.
	Pallavicinia levieri Schiffn.
	Radula complanata (L.) Dumort.
	Diphyscium longifolium Griff.
	Metzgeria furcata (L.) Corda
	Heteroscyphus coalitus (Hook.) Schiffn.
	Acroporium lamprophyllum Mitt.
1100	*Leucobryum bowringii* Mitt.
	Bazzania semiopacea N. Kitag.
	Thuidium glaucinoides Broth.
	Heteroscyphus zollingeri (Gottsche) Schiffn.
	Drepanolejeunea dactylophora (Gottsche Lindenb. & Nees) J. B. Jack & Steph.
	Pylaisiadelpha yokohamae (Broth.) W. R. Buck
	Neolepidozia wallichiana (Gottsche) Fulford & J. Taylor
	Pseudotaxiphyllum pohliaecarpum (Sull. & Lesq.) Z. Iwats.
	Calypogeia arguta Nees & Mont.
	Heteroscyphus argutus (Reinw. Blume & Nees) Schiffn.
	Pallavicinia levieri Schiffn.
	Radula complanata (L.) Dumort.
	Diphyscium longifolium Griff.
	Metzgeria furcata (L.) Corda
	Heteroscyphus coalitus (Hook.) Schiffn.
	Acroporium lamprophyllum Mitt.
1200	*Leucobryum bowringii* Mitt.
	Bazzania semiopacea N. Kitag.
	Thuidium glaucinoides Broth.
	Heteroscyphus zollingeri (Gottsche) Schiffn.
	Drepanolejeunea dactylophora (Gottsche Lindenb. & Nees) J. B. Jack & Steph.
	Pylaisiadelpha yokohamae (Broth.) W. R. Buck
	Neolepidozia wallichiana (Gottsche) Fulford & J. Taylor
	Calypogeia arguta Nees & Mont.
	Heteroscyphus argutus (Reinw. Blume & Nees) Schiffn.
	Pallavicinia levieri Schiffn.
	Radula complanata (L.) Dumort.
	Diphyscium longifolium Griff.
	Metzgeria furcata (L.) Corda
	Heteroscyphus coalitus (Hook.) Schiffn.
1300	*Leucobryum bowringii* Mitt.
	Bazzania semiopacea N. Kitag.
	Thuidium glaucinoides Broth.

	Heteroscyphus zollingeri (Gottsche) Schiffn.
	Drepanolejeunea dactylophora (Gottsche Lindenb. & Nees) J. B. Jack & Steph.
	Pylaisiadelpha yokohamae (Broth.) W. R. Buck
	Neolepidozia wallichiana (Gottsche) Fulford & J. Taylor
	Pseudotaxiphyllum pohliaecarpum (Sull. & Lesq.) Z. Iwats.
	Heteroscyphus argutus (Reinw. Blume & Nees) Schiffn.
	Pallavicinia levieri Schiffn.
	Radula complanata (L.) Dumort.
	Diphyscium longifolium Griff.
	Metzgeria furcata (L.) Corda
	Heteroscyphus coalitus (Hook.) Schiffn.
	Acroporium lamprophyllum Mitt.
1400	*Leucobryum bowringii* Mitt.
	Bazzania semiopacea N. Kitag.
	Thuidium glaucinoides Broth.
	Heteroscyphus zollingeri (Gottsche) Schiffn.
	Drepanolejeunea dactylophora (Gottsche Lindenb. & Nees) J. B. Jack & Steph.
	Pylaisiadelpha yokohamae (Broth.) W. R. Buck
	Neolepidozia wallichiana (Gottsche) Fulford & J. Taylor
	Pseudotaxiphyllum pohliaecarpum (Sull. & Lesq.) Z. Iwats.
	Calypogeia arguta Nees & Mont.
	Heteroscyphus argutus (Reinw. Blume & Nees) Schiffn.
	Radula complanata (L.) Dumort.
	Diphyscium longifolium Griff.
	Metzgeria furcata (L.) Corda
	Brotherella erythrocaulis (Mitt.) M. Fleisch.
	Heteroscyphus coalitus (Hook.) Schiffn.
	Acroporium lamprophyllum Mitt.
